# Evaluation of resident attitudes and self-reported competencies in health advocacy

**DOI:** 10.1186/1472-6920-10-82

**Published:** 2010-11-18

**Authors:** Sara Stafford, Tara Sedlak, Mark C Fok, Roger Y Wong

**Affiliations:** 1 Fellow, Division of Endocrinology, Department of Medicine, University of British Columbia, Vancouver, Canada; 2 Fellow, Division of Cardiology, Department of Medicine, University of British Columbia, Vancouver, Canada; 3 Chief Medical Resident, Department of Medicine, University of British Columbia, Vancouver, Canada; 4 Clinical Professor, Division of Geriatric Medicine, Department of Medicine, University of British Columbia, Vancouver, Canada

## Abstract

**Background:**

The CanMEDS Health Advocate role, one of seven roles mandated by the Royal College of Physicians and Surgeons Canada, pertains to a physician's responsibility to use their expertise and influence to advance the wellbeing of patients, communities, and populations. We conducted our study to examine resident attitudes and self-reported competencies related to health advocacy, due to limited information in the literature on this topic.

**Methods:**

We conducted a pilot experience with seven internal medicine residents participating in a community health promotion event. The residents provided narrative feedback after the event and the information was used to generate items for a health advocacy survey. Face validity was established by having the same residents review the survey. Content validity was established by inviting an expert physician panel to review the survey. The refined survey was then distributed to a cohort of core Internal Medicine residents electronically after attendance at an academic retreat teaching residents about advocacy through didactic sessions.

**Results:**

The survey was completed by 76 residents with a response rate of 68%. The majority agreed to accept an advocacy role for societal health needs beyond caring for individual patients. Most confirmed their ability to identify health determinants and reaffirmed the inherent requirements for health advocacy. While involvement in health advocacy was common during high school and undergraduate studies, 76% of residents reported no current engagement in advocacy activity, and 36% were undecided if they would engage in advocacy during their remaining time as residents, fellows or staff. The common barriers reported were insufficient time, rest and stress.

**Conclusions:**

Medical residents endorsed the role of health advocate and reported proficiency in determining the medical and bio-psychosocial determinants of individuals and communities. Few residents, however, were actively involved in health advocacy beyond an individual level during residency due to multiple barriers. Further studies should address these barriers to advocacy and identify the reasons for the discordance we found between advocacy endorsement and lack of engagement.

## Background

The CanMEDS framework was designed as an innovative framework for medical education with the goal of ensuring the highest standards and quality of health care [1]. It was first approved by the governing council of the Royal College of the Physicians and Surgeons of Canada (RCPSC) in 1996, and subsequently revised with a new edition, *CanMEDS 2005 *[1,2]. This framework has since been incorporated into the objectives for residency training, examinations, and program accreditation across Canada for all specialty post-graduate medical education programs accredited by the Royal College of Physicians and Surgeons.

There are seven CanMEDS roles, each outlining key physician competencies to address the multi-faceted roles that physicians play. The central role is that of a medical expert, while the six other roles include that of a communicator, collaborator, manager, health advocate, scholar, and professional. To date, teaching and evaluating the six non-medical expert roles has been challenging. In 2003, at the annual meeting of the Association of Canadian Medical Colleges in Quebec City, Frank *et al*. described the results of a 2001 RCPSC survey [3]. They reported that significant progress had been made in implementing the CanMEDS framework in Canada, but that the roles of manager and health advocate were the most difficult roles to teach and evaluate. In 2006, recent graduates of Canadian general internal medicine (GIM) programs were surveyed to determine whether they felt well prepared in different aspects of medicine for their future careers [4]. In general, most graduates felt well prepared in the role of medical expert and over half felt adequately taught in the CanMEDS roles of communicator, collaborator, scholar, and professional. However, graduates felt inadequately prepared in the roles of health advocate and manager.

The role of a health advocate, as per the CanMEDS 2005 framework, is defined as "physicians (who) responsibly use their expertise and influence to advance the health and well-being of individual patients, communities, and populations."[5] The key competencies outlined in this framework are that physicians are able to: respond to individual patient health needs and issues as part of patient care; to respond to the health needs of the communities that they serve; to identify the determinants of health of the populations that they serve; and to promote the health of individual patients, communities and populations [5]. Similarly, the American Medical Association declaration of professional responsibility states that physicians must "advocate for the social, economic, educational, and political changes that ameliorate suffering and contribute to human well-being."[6] Despite these definitions and endorsements, teaching health advocacy is challenging. Advocacy has a broad scope and practice, thus contributing to a lack of clarity on expectations by teachers and learners alike. Advocacy often blurs the roles of professional, communicator and manager due to overlapping competencies, thus perhaps making its role less prominent for some. Lastly, many residency programs lack a distinct curriculum on advocacy due to a lack of recognition of this role, and difficulty in evaluating competency.

A review of the literature on resident attitudes towards health advocacy revealed few published studies [7,8]. One study in the United States described a community-based advocacy training program for pediatric residents, where residents were assigned in a longitudinal manner to a community collaborative in their first year and eventually implemented a project in their last year of residency [7]. It was felt that resident attitudes toward community-centered advocacy were noticeably positive within the program, but no qualitative data was provided [7]. A Canadian study explored the views of faculty and residents about teaching and evaluating health advocacy in focus groups, and found that residents required clarity on the definition of health advocate, and that many barriers to learning and teaching health advocacy exist. However, residents acknowledged a health-advocate role as part of their social responsibility to society as a physician [8].

To our knowledge, resident viewpoints regarding health advocacy are poorly understood and self-reported competencies are unknown. The objective of our project was therefore to describe resident attitudes and self-reported competencies towards the role of health advocate.

## Methods

### Study population

This study was conducted in 2008 at the University of British Columbia (UBC) in Vancouver, British Columbia, Canada. The UBC internal medicine residency program has 111 residents in training with 43 post graduate year (PGY)-1, 33 PGY-2, and 35 PGY-3 residents. We received approval from the UBC internal medicine residency training committee to implement the study and disseminate its results. We told all residents that by responding to the survey, they have granted their verbal consent for us to analyze and report our findings in group data format.

### Survey design

We conducted a pilot health advocacy experience in January 2008. Specifically, seven residents from the UBC internal medicine residency program volunteered to take part in a community health promotion event in Vancouver's downtown eastside, an area infamous in the city for poverty, crime and homelessness. This area also includes Chinatown and the residents were involved in performing blood pressure measurements and provided cardiovascular risk factor counseling to 200 Cantonese-speaking community-dwelling older adults. Subsequently, these residents provided narrative feedback regarding their experience and possible barriers to engaging in future health advocacy roles. The information gathered was then used to generate the 20 items of the pilot Health Advocacy Questionnaire. Fourteen items were developed with 5-point Likert scale responses, 4 items with yes/no/undecided responses, and 2 items with open-ended responses. Face validity of the questionnaire was established by having the same 7 residents who participated in the advocacy experience review the survey. Content validity was established by inviting an expert physician panel to review the survey. The expert panel consisted of 4 internists with extensive experience in the health advocate role by means of being involved in patient advocacy at the individual, population and societal levels. The panel consisted of a rural community internist, an urban community internist, an academic internist, and an internist involved in international health. After the development of the Health Advocacy Questionnaire, the UBC internal medicine program held a weekend academic retreat for all UBC internal medicine residents focusing on health advocacy. The speakers at this weekend retreat included physicians, a pharmacist and a lawyer who spoke about their local and international experiences working with Doctors Without Borders, various HIV initiatives in Africa, the economics of drug coverage and medico-legal advocacy. The Health Advocacy Questionnaire was distributed to all internal medicine residents at the end of the "Advocacy in Action" retreat. (Table 1).

**Table 1:** Health Advocacy Questionnaire

	Strongly Disagree	Disagree	Neutral	Agree	Strongly Agree
1. It is part of my job as an internist to advocate for populations' health needs within society.	1	2	3	4	5

2. I feel that my role as a health advocate extends beyond the individual patient(s) I am treating.	1	2	3	4	5

3. I am able to identify the health needs of an individual patient during patient care (beyond biomedical needs).	1	2	3	4	5

4. I can describe the health needs of the communities that I serve.	1	2	3	4	5

5. I can identify the determinants of health (psychological, biological, social, cultural and economic aspects) of patients in my community.	1	2	3	4	5

6. I can describe how public policy impacts on the health of populations that I serve.	1	2	3	4	5

7. I can describe the requirements inherent in health advocacy (e.g. altruism, social justice, autonomy, integrity and idealism), as described by the Royal College of Physicians and Surgeons of Canada.	1	2	3	4	5

8. I understand the opportunities available for internists to function as health advocates.	1	2	3	4	5

9. I am able to help my patient(s) navigate the health care system.	1	2	3	4	5

10. My current competence in being a health advocate has increased compared to 1 month ago.	1	2	3	4	5

11. My current knowledge about health advocacy has increased compared to 1 month ago.	1	2	3	4	5

12. Compared to 1 month ago, I feel more able to practice health advocacy in ways I would not have otherwise done during a regular clinical rotation.	1	2	3	4	5

13. The current likelihood of my engaging in health advocacy activity/activities has increased compared to 1 month ago.	1	2	3	4	5

14. I am more likely to recommend health advocacy activity/activities to others compared to 1 month ago.	1	2	3	4	5

15. I engage(d) in health advocacy activities during (check all that apply):					
__ High school	__ Medical school				
__ University/College	__ Residency				
__ Graduate studies					
16. I am currently engaging in health advocacy activities	__Yes	__ No			
17. I plan to engage in health advocacy activities					
a. in my remaining time as a resident	__Yes	__ No	__ Undecided		
b. in fellowship	__Yes	__ No	__ Undecided		
c. post-fellowship	__Yes	__ No	__ Undecided		
If Yes to 17 a), b), or c), please answer the following two optional questions.					
What population(s) or patient group(s) would benefit from your advocacy efforts?					
How would you hope to see them benefit, in both the short and long term?					
18. Comments?					

### Data analysis

For items with Likert responses, data is presented as a percentage of respondents who chose 1 (strongly disagree), 2 (disagree), 3 (neutral), 4 (agree) or 5 (strongly agree). Percentages were reported for other items. For the open-ended items, respondents' comments were analyzed for common themes by thematic content analysis using the Atlas.ti software (version 6.0, 2009).

## Results

The survey was completed by 76 of 111 residents for a response rate of 68%. Thirty-two completed responses came from the PGY-1 year (42%), 22 (29%) from the PGY-2 year and 22 (29%) from the PGY-3 year.

### Baseline attitudes and self-reported competencies in health advocacy

**Figure 1:**
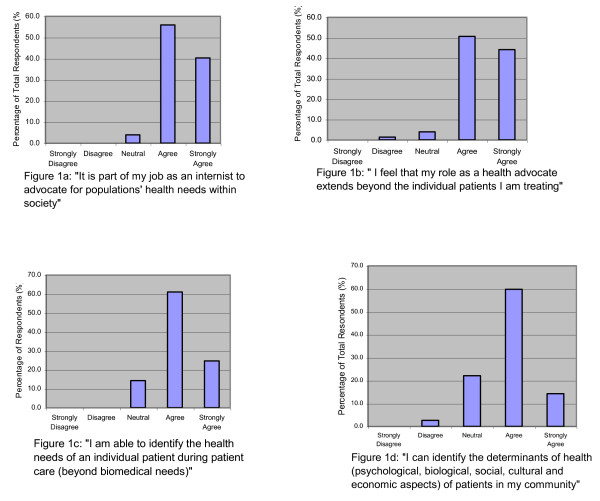
**Baseline attitudes and self-reported competencies in health advocacy**.

The majority of residents who responded agreed to accept an advocacy role for societal health needs beyond caring for individual patients. Specifically, 96% of residents agreed or strongly agreed that it was part of their job to advocate for populations' health needs within society (Figure 1a). Ninety-five percent of respondents felt their role as a health advocate extended beyond the individual patient(s) they were treating (Figure 1b). Further, most residents self reported their ability to identify health determinants and reaffirmed the inherent requirements for health advocacy. Eighty-six percent of residents agreed or strongly agreed that they could identify the health needs of an individual patient (Figure 1c) while 75% said they could identify the determinants of health of (psychological, biological, social, cultural, economic aspects) of patients in their community (Figure 1d). Multiple target populations were identified as potential beneficiaries: geriatric populations, minority or special populations (such as ethnic groups, women, immigrants, and patients of lower socioeconomic status), and international communities.

### Engagement in health advocacy activities

**Figure 2:**
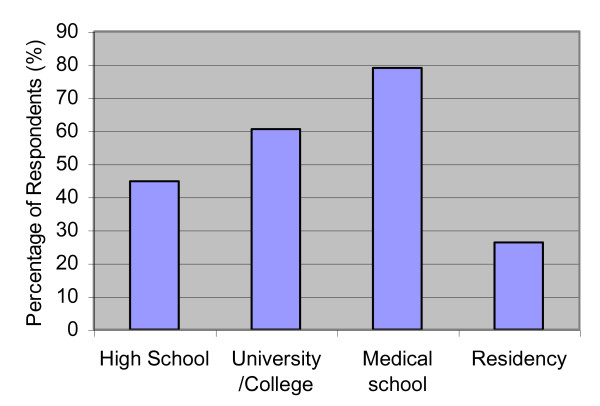
**"I engaged in health advocacy during..."**.

**Figure 3:**
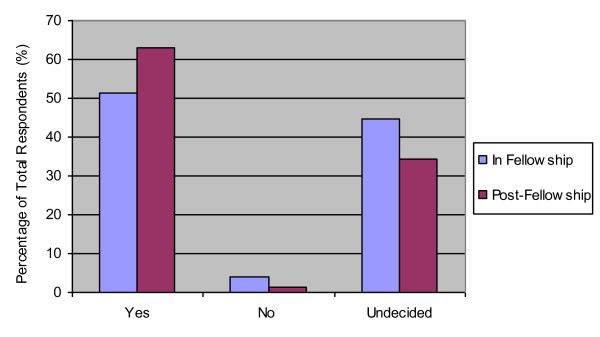
**"I plan to engage in health advocacy activities during fellowship or post-fellowship"**.

Seventy-four percent of residents reported no current engagement in advocacy activity. There was increased reporting of participation or engagement in health advocacy activities from high school to university to medical school; however, a sharp drop-off in participation occurred during residency training (Figure 2). Forty-five and thirty-four percent of residents said they were undecided as to whether they would engage in advocacy during their future fellowship or post-fellowship, respectively (Figure 3).

### Perceived barriers to engagement in health advocacy

A free text section for "Comments" at the end of the questionnaire generated several comments about common barriers to advocacy engagement. These included limited time, limited rest, and excess stress during residency training. Some of the sample comments from respondents regarding these topics were as follows:

"Physicians need time and rest. We need somebody to advocate for our needs before we can advocate for the patients that we serve.... We're expected to give and give..."

"... My main barrier to doing more advocacy is time, given all our other requirements for clinical learning, research, and clinical service"

"Residency is a very stressful period...."

## Discussion

We believe this is the first study of its kind that surveyed Canadian internal medicine residents to examine their attitudes and self-reported competencies in health advocacy. Our results demonstrate that residents generally agreed that the role of health advocate is an important aspect of an internal medicine specialist, and that this role extends beyond looking after the individual patient. Residents felt that they possessed the main competencies in health advocacy. Specifically, they believed that they were able to identify the health needs of the individual patient beyond their biomedical needs, and the determinants of health within their community (psychological, biological, social, cultural, and economic aspects). These are important aspects to recognize because they suggest an awareness of the significance of the health advocate role, as well as an ability to carry out this role in society.

Despite this endorsement of the importance of health advocacy, and the related self-reported competencies, there was a marked deficit of self-reported engagement in health advocacy activities within the resident cohort. Seventy-four percent of residents stated that they were not currently engaging in health advocacy activities; this is even more striking when compared to the prior engagement of this cohort of residents earlier in their education. While it is possible that residents might not have identified/recognized advocacy activities that they were already participating in during residency and labeled them accordingly, there were likely other systematic reasons. In the free text "Comments" section, residents frequently spoke of perceived barriers to health advocacy engagement. The themes of lack of time, insufficient rest, and the high-stress environment of residency were predominant. Such comments are not surprising given the competing interests of educational commitments, frequent on call-shifts and long hours in demanding residencies, all of which contribute to resident stress [9-11]. In one of the few prior publications of advocacy education, one resident noted that "the clinical time demands of residency are often a significant barrier to resident participation in the community activities."[7]

Interestingly, a large proportion of residents were undecided as to whether they would engage in health advocacy in the future during their fellowship or independent practice. This discordance between the endorsement of the role of health advocacy and the lack of current and possibly future participation is a surprising finding, especially in light of residents' self-reported competency in this area and their prior engagement in health advocacy activities. One possible reason may be time constraints and or the lack of remuneration for participating in such events. Other hypotheses that have been suggested include a gradual erosion of altruism during residency resulting in poor engagement, and endorsement of advocacy because it is socially desirable, but not inherently believed [12]. Such hypotheses need to be explored in further studies.

There were several limitations in our study. We used self-assessed competence as a surrogate for determining resident competency in the role of health advocate, but self-assessment has never been validated in the literature for this purpose. We also did not have data from non-respondents, and therefore could not comment on any possible differences between respondents and non-respondents. Future development of a gold-standard for assessing competency in the role of health advocate is needed to determine the validity of self-reporting in this non-medical expert role. Additionally, we asked residents to recall their prior engagement in health advocacy activities. This method is susceptible to recall bias, and hence it would be informative to survey a matched cohort of students during earlier stages of medical training in order to quantify engagement at those times.

Health advocacy has reached a critical junction in medical education. If it is to be a core competency role for practicing physicians, attitudes towards advocacy need to be examined, its teaching be emphasized, its practice be adopted and its evaluation be more clearly defined by medical educators and their institutions. Our study of resident attitudes and competencies suggests endorsement of advocacy as a principle and self-reported competency, but limited engagement in present and possibly future advocacy events. Based on our data, we recommend the following steps: Residents reported limited time as one of the barriers for engaging in health advocacy. Thus, allowing dedicated time to engage in health advocacy projects within residency training in a longitudinal manner should be explored. Future studies could be also be directed toward performing a time audit to further quantify the time required to engage in health advocacy activities, and to determine if there are mechanisms by which the time required for participation can be limited such that it is perceived to be less of a barrier to health advocacy engagement. Residents also reported excessive stress during the training period as being a significant deterrent to health advocacy engagement. Health advocacy activities should include health care providers amongst the targeted populations, focusing on stress management as this is known to be a significant issue within the profession. It would be valuable to determine the contributors to perceived stress during the residency training period, in order to target these issues. Teaching and curriculum advancement in advocacy needs further development and methods of assessment need to be evaluated. Finally, we identified certain population groups "at risk" through the questionnaire: geriatric populations, people with a lower socio-economic status, women in poverty and minority populations. These marginalized groups should be approached to develop potential community advocacy projects on a long-term basis.

## Conclusions

Resident attitudes towards advocacy were generally positive with recognition of the need to advocate beyond the level of patients to that of society. Residents felt competent identifying determinants of health; however, few residents were actually engaging in advocacy events. Further studies should address barriers to advocacy and identify the reasons for the discordance we found between advocacy endorsement and lack of engagement.

## Competing interests

The authors declare that they have no competing interests.

## Authors' contributions

RYW conceived of the study. SS, TS, MCF and RYW participated in its design and coordination, and helped draft the manuscript. All authors read and approved the final manuscript.

## Pre-publication history

The pre-publication history for this paper can be accessed here:

http://www.biomedcentral.com/1472-6920/10/82/prepub
